# Enhancement of sciatic nerve regeneration with dual delivery of vascular endothelial growth factor and nerve growth factor genes

**DOI:** 10.1186/s12951-020-00606-5

**Published:** 2020-03-14

**Authors:** Zhiwei Fang, Xuemei Ge, Xuan Chen, Yang Xu, Wei-En Yuan, Yuanming Ouyang

**Affiliations:** 1 Department of Orthopedics, Shanghai Jiao Tong University Affiliated Sixth People’s Hospital, Shanghai, 200233 China; 2 School of Light Industry and Food Engineering, Nanjing Forestry University, Nanjing, 210037 China; 3 Engineering Research Center of Cell & Therapeutic Antibody, Ministry of Education, and School of Pharmacy, Shanghai Jiao Tong University, Shanghai, 200240 China; 4 Shanghai Sixth People’s Hospital East Affiliated to Shanghai University of Medicine & Health Sciences, Shanghai, 201306 China

**Keywords:** Sciatic nerve injury, Autologous nerve grafting, Vascular endothelial growth factor (VEGF), Nerve growth factor (NGF), Gene therapy

## Abstract

**Background:**

Peripheral nerve injury is one common clinical disease worldwide, in which sciatic nerve is anatomically the most challenging to regenerate given its length and large cross-sectional area. For the present, autologous nerve grafting remains to be the most ideal strategy when treating with sciatic nerve injury. However, this method sacrifices healthy nerves and requires highly intensive surgery, still calling for other advanced alternatives for nerve grafting.

**Results:**

In this study, we utilized previously well-established gene delivery system to dually deliver plasmid DNA (pDNA) encoding vascular endothelial growth factor (VEGF) and nerve growth factor (NGF), exploring therapeutics for sciatic nerve injury. Low-molecular-weight branched polyethylenimine (bPEI) was constructed as the backbone structure of gene vectors, and it was further crosslinked to synthesize degradable polycations via the conjugation of dialdehydes. Potential synergistic effect between VEGF and NGF proteins were observed on rat sciatic nerve crush injury model in this study.

**Conclusions:**

We concluded that dual delivery of plasmid VEGF and NGF as gene therapy could enhance sciatic nerve regeneration.

## Background

Causing physical disability among young males, peripheral nerve injury is clinically a main contributor for reduced functional capacity [1, 2]. Usually peripheral nerve injury comes with deficits in sensation, loss of muscle power, or a combination of both. This disease brings much inconvenience to patients and poses an enormous financial burden to the whole society. In a retrospective study, it was reported that it cost at least 150 billion dollars annually for the treatment of peripheral nerve injury in United States [3], of which the reason was thought to be the prolonged rehabilitation and absenteeism of this disease [2].

Current methods for peripheral nerve regeneration includes self-healing, surgery treatment, and physical therapy, usually supplemented with other treatments focusing on reducing pain and discomfort of patients. Among them, autologous nerve grafting remains to be the gold standard treatment for severe sciatic nerve regeneration [1, 4–6]. However, problems of low success rate of surgery, limited supply of donor nerves, insufficiency in large nerve gaps and inherent damage to donor sites have posed a huge challenge to the clinical use of autologous nerve grafting [6, 7]. Currently, recovery with tissue-engineered methods is gradually becoming an alternative strategy for sciatic nerve reconstruction [8–10]. However, after having an overview of reported studies on tissue-engineered nerve grafting, we found that most pre-clinical researches focused on the role of neurogenesis in the peripheral nerve recovery, while the angiogenesis effect of blood vessels were often ignored [11]. For now, we should realize that nerve regeneration is a complicated system that involves delicate balance of molecular and tissue signals, in which vascularization should be indispensable. It was found in several studies that the regeneration of endoneural vasculature often precedes the outgrowth of axons [12–15]. With the growth and regeneration of peripheral nerve, metabolic demands are urgently increasing, in which more oxygen and nutrients are needed for the supply, and more waste products need to be removed. In this case, enough blood vessels are of much essence, to exchange nutrients and waste products with outer environment and maintain the viability of new regenerative nerves [16–19]. Besides that, impressive comparisons in distribution pattern between the nervous and vascular network were made in several studies [12, 20, 21]. It was found that parallels exist between vessels and nerves and they tracked together towards the targets. A recent bioinformatic study analyzing transcription molecules suggested that angiogenesis and peripheral nerve regeneration may share some common biological pathways or networks after the transection of sciatic nerve [22]. Consistent results were also reported in another study that common genetic pathways were shared such as that of netrin-1 [23]. Therefore, a hypothesis could be made that both revascularization and neurogenesis are significantly important for the regeneration and reconstruction of sciatic nerve injury [22, 24].

Previously, we have reported the establishment of a novel and efficient gene delivery system that focused on the synthesis of polycationic materials as gene vectors [25, 26]. Branched polyethylenimine (bPEI) with low molecule weight (1.8 kDa) was selected as the backbone of gene vectors and was then conjugated with dialdehydes, 2,6-pyridinedicarboxaldehyde (PDA). PDAPEI was finally synthesized to be with both high transfection efficiency and low cell cytotoxicity. This synthesized material could tightly wrap plasmid DNA (pDNA) into uniform spherical nanoparticles, transfect pDNA into the cells with high efficiency and relative low toxicity [25, 26]. In this study, we utilized the system to simultaneously deliver genes encoding vascular endothelial growth factor (VEGF) and nerve growth factor (NGF) for the first time [27–29]. We expected the combination of neuronal regeneration from NGF expression and neovascularization boosted by VEGF protein could lead to the improvement of nerve healing. To confirm that, we investigated the gene therapy system on rat Schwann cells (RSC) and further established a sciatic nerve crush injury model on Sprague–Dawley (SD) rats. Functional and morphological assessment were evaluated in vivo, of which the results indicated a satisfactory recovery of sciatic nerves with increased thickness of myelinated fibers. Besides, the density of neomicrovessels also increased in rats, implying potential benefits in neovascularization. All of these results confirmed the great therapeutic potential in enhancement of sciatic nerve regeneration with dual delivery of VEGF and NGF genes.

## Results

### Characterizations of PDAPEI/pDNA polyplexes

#### Particle size and zeta potential

Both particle size and zeta potential are critical physicochemical properties of polyplexes. They are not only good indicators of condensing performance of polyplexes, but also important in cell endocytosis and gene release. Generally, particle size ranging from 100 to 300 nm is an ideal choice for delivery of nanoparticles, and as for zeta potential, polyplexes with positive-charge are usually expected. Particle size of PDAPEI/pDNA was previously reported [26]. Particle sizes stabilized around 125 nm and there were no significant differences observed in particle size among polyplexes at different ratios, which indicates a robust and stable system. While for zeta potential of PDAPEI/pDNA polyplexes was around 30 mV also confirmed the stable system we constructed [26].

#### Morphology observation of PDAPEI/pDNA polyplexes

**Fig. 1:**
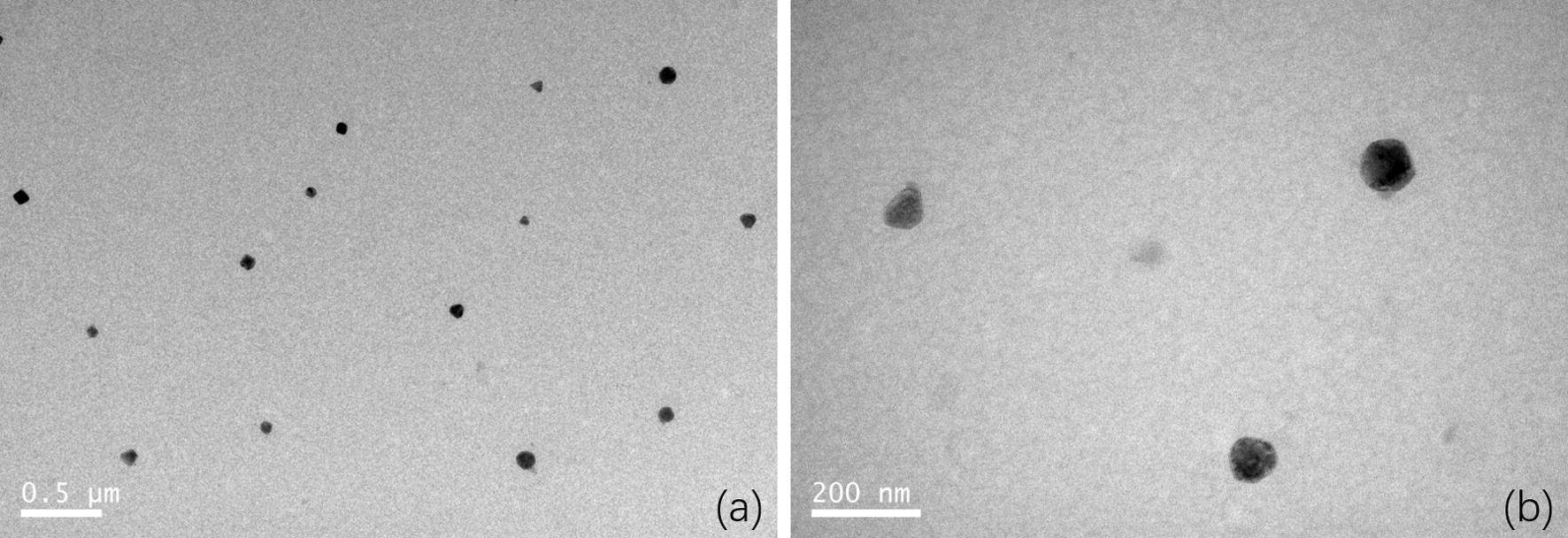
Transmission electron microscopy (TEM) images of PDAPEI/pDNA polyplexes at 30 w/w ratio. The scale bars are **a** 0.5 μm, and **b** 200 nm, respectively

Transmission electron microscopy (TEM) images of PDAPEI/pDNA polyplexes at 30 w/w ratio were demonstrated in Fig. 1. Polyplexes were spherical and distributed evenly in the system. The result was consistent with our expectations. Interestingly, it was found in a comprehensive study that worm-like nanoparticles can have a more enhanced gene delivery efficiency in vivo compared with rod-like nanoparticles and nanospheres [30]. This indicates the importance of polyplexes shape in cellular uptake and transport.

### In vitro cell cytotoxicity of PDAPEI/pDNA polyplexes

**Fig. 2:**
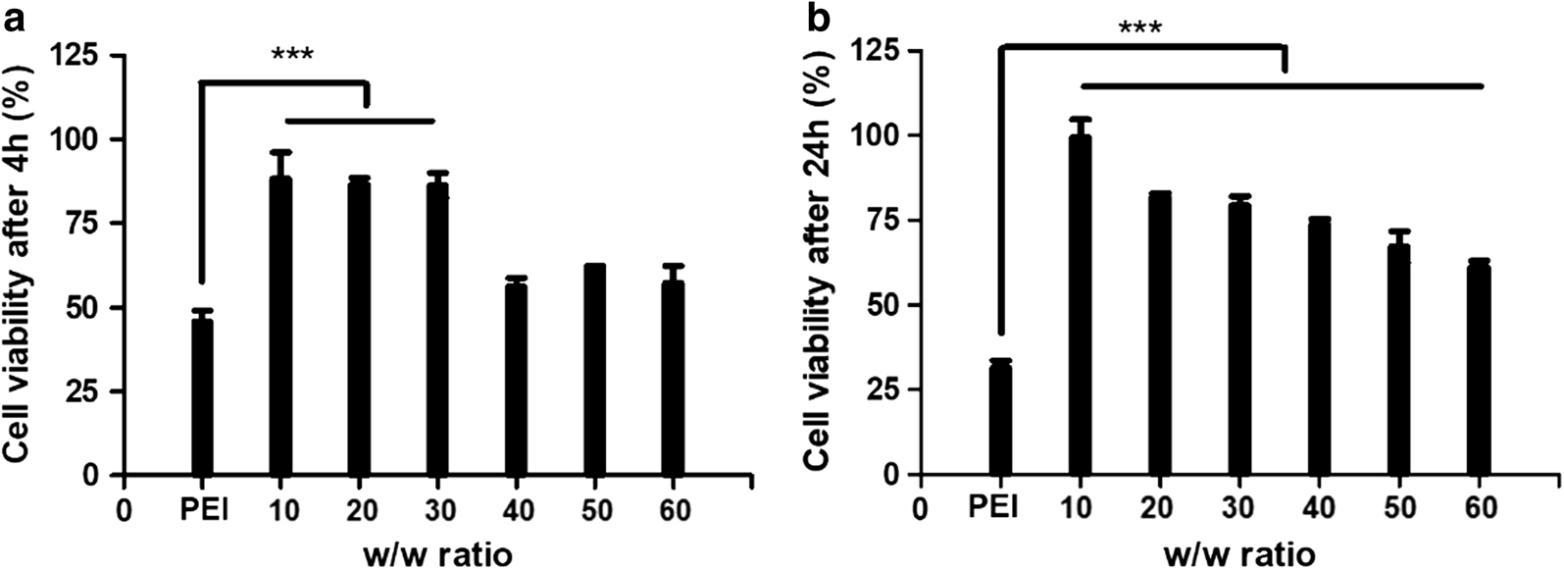
In vitro cell cytotoxicity of PDAPEI polyplexes at 10, 20, 30, 40, 50 and 60 w/w ratios after **a** 4 h; **b** 24 h. ***Indicates p < 0.005; data are mean ± SD (n = 6)

The cell cytotoxicity of PDAPEI/pDNA polyplexes on rat Schwann cells (RSC) were evaluated by CCK-8 reagent and the results were shown in Fig. 2. In general, cell viability of PDAPEI/pDNA polyplexes was higher than that of PEI (25 kDa)/pDNA polyplex group (***p < 0.005). With the increase of w/w ratio, cell viability decreased gradually in 24-h test. Compared to 4-h treatment, cell viability of PEI (25 kDa)/pDNA polyplex group decreased dramatically in the 24-h treatment, which may due to the non-degradability of PEI (25 kDa) in cells. While for PDAPEI/pDNA polyplexes, no such phenomenon could be observed. Cell viability in 24-h test here seemed to be even higher than that in 4-h test, especially at high w/w ratios (Fig. 2). This unexpected results may come from the calculation method of CCK-8 measurement. The cell viability we got was a relative value, with the viability of cells treated with PBS (keep the same volume with each sample group) and cultured in DMEM complete growth medium as reference. In 4-h test, cells in polyplexes group may receive an instant attack since polyplexes did not degrade in the beginning, and thus the cell viability was much lower compared to that in PBS group. While in the 24-h test, most polyplexes may degrade and thus the effect of cell damage stopped. Meanwhile, cells in PBS group may die for lack of enough supply of nutrient. In this case, the relative value of cell viability could be higher.

### In vitro cell transfection

**Fig. 3:**
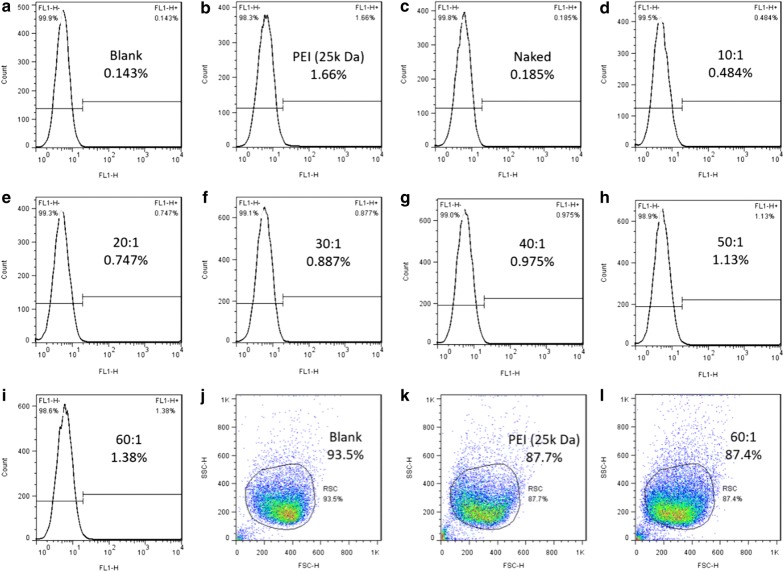
Flow cytometry analysis of PDAPEI/pDNA polyplexes at various ratios. Images **a**–**i** demonstrate transfection efficiency of blank group (PBS), positive group (PEI 25 kDa), PDAPEI/pDNA group at 10, 20, 30, 40, 50 and 60 w/w ratios, respectively. Images **j**–**l** demonstrate status of cells

Cells transfected with PDAPEI/pDNA polyplexes were analyzed by flow cytometer to determine the expression of GFP. As is demonstrated in Fig. 3, there were almost no GFP expression detected in PBS blank control group (0.143%) and naked group (0.185%). While for the positive control, the transfection efficiency is relatively high (PEI 25 kDa, 1.66%). As for cells treated with PDAPEI/pDNA polyplexes at various ratios, the transfection efficiency differed. And we could observe a positive correlation between the transfection efficiency and PDAPEI/pDNA w/w ratio. With the increase of w/w ratio, the amount of PDAPEI in the formulation increased accordingly, and the transfection efficiency become higher (0.484% at 10 w/w ratio to 1.38% at 60 w/w ratio). This suggested that with more PDAPEI in the formulation, more gene could be successfully delivered into the cell. We could further deduce that if more PDAPEI was added in the polyplexes, it was possible that the transfection efficiency could be higher. However, apart from transfection efficiency, other significant factors like cell cytotoxicity should also be taken into consideration, thus it was impractical to over-emphasize the role of transfection efficiency here in gene delivery study. Combining all these results above, we finally adopted PDAPEI/pDNA at 30 w/w ratio as our optimal condition to use in the following experiments.

### In vitro neural expression and RSC morphology observation

**Fig. 4:**
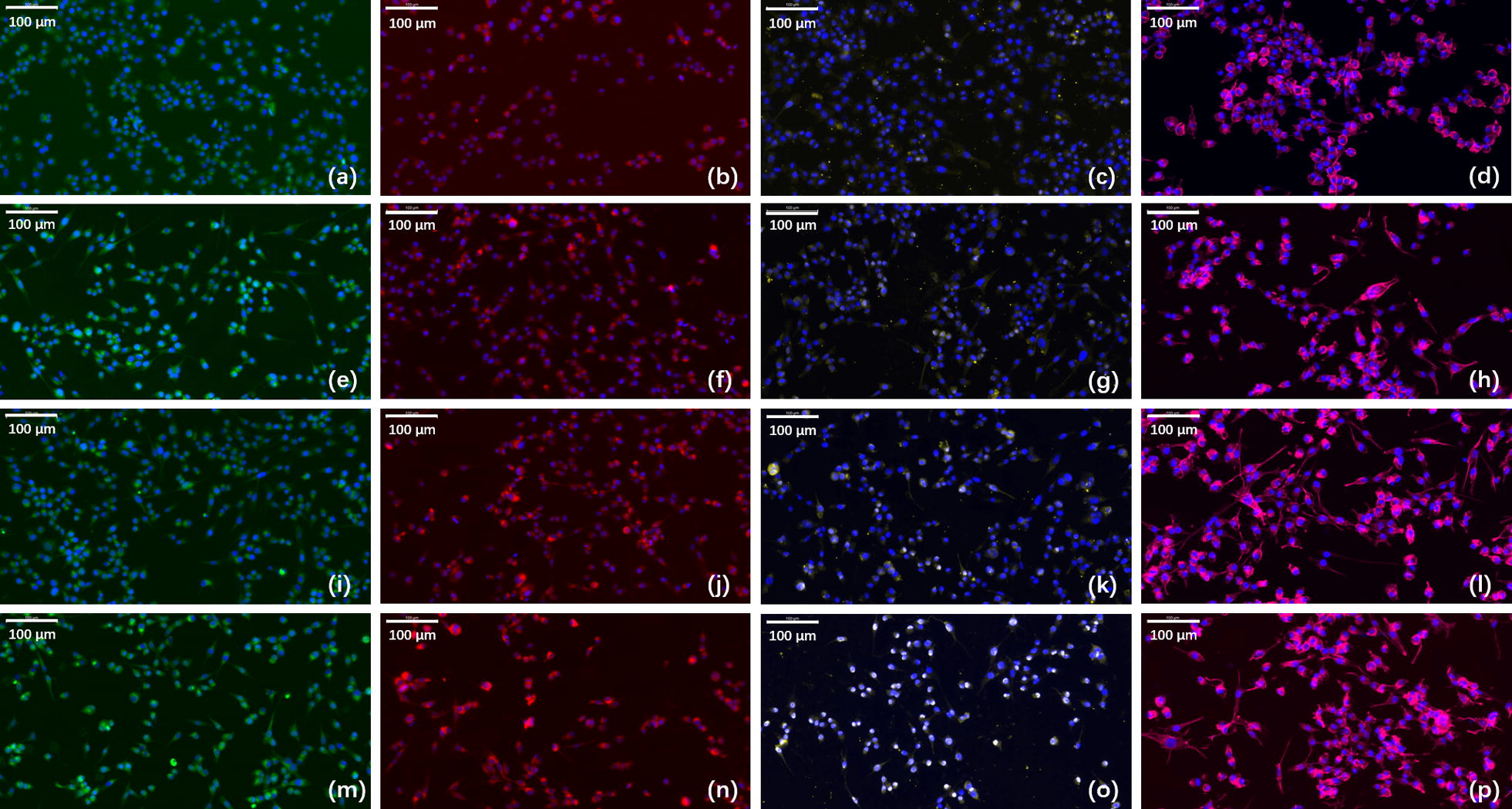
Immunofluorescence staining for GFAP, S100, and Tuj1 and phalloidin staining for RSC morphology observation in **a**–**d** PBS treated group, **e**–**h** VEGF group, **i**–**l** NGF group, and **m**–**p** VEGF + NGF group. Images **a**, **e**, **i**, **m** are staining for GFAP (green) and DAPI (blue). Images **b**, **f**, **j**, **n** are staining for S100 (red) and DAPI (blue). Images **c**, **g**, **k**, **o** are staining for Tuj1 (yellow) and DAPI (blue). Images **d**, **h**, **l**, **p** are staining for actin (pink) and DAPI (blue). Scale bar is 100 μm

Immunofluorescence assay were used to evaluate expression levels of glial fibrillary acidic protein (GFAP), S100, and Class III β-tubulin (Tuj1) of cells transfected with PDAPEI/pDNA polyplexes (Fig. 4). GFAP is an intermediate filament protein and expressed in many cell types in the central and peripheral nervous system. S100 is small protein with molecular weight of 10–12 kDa and is the most used SC markers in the field. Tuj1 has been a classic marker of neurons in the central and peripheral nervous system and can differentiate neurons from glial cells. As can be seen in Fig. 4m–o, higher expression of GFAP, S100 and Tuj1 was observed in VEGF + NGF group, which indicates a promoting effect on neural expression and differentiation with the combination of VEGF and NGF.

Phalloidin staining was performed as well to observe the actin filament of RSCs cultured in different conditions (Fig. 4d, h, l, p). More protrusions were observed in VEGF + NGF group, which indicates a good condition for RSC growth. Moreover, actin polymerization and organization is believed to be related to RSC migration, and repair and regeneration of injured peripheral nerves would be hindered if the actin cytoskeleton was limited [31]. Therefore, we could draw a conclusion that combinational delivery of VEGF and NGF gene could boost the growth and functional behavior of RSCs.

### Peripheral sciatic nerve crush injury model and functional analysis

**Fig. 5:**
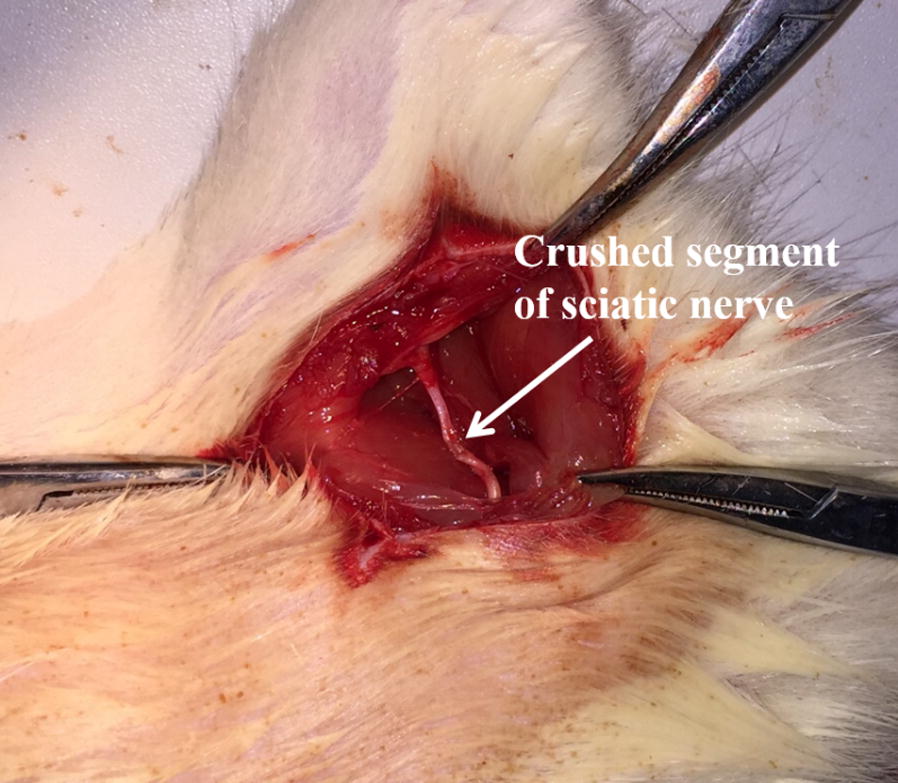
Illustration of peripheral sciatic nerve crush injury model

As shown in Fig. 5, after hemostats being removed, the crushed segment of sciatic nerve become translucent, while other parts remained undamaged. After 4 weeks, all rats were ready for further analysis. Before the sacrifice, walking track analysis was performed to evaluate the regeneration and functional recovery of peripheral nerve. According to the instructions demonstrated in existing studies, sciatic function index (SFI), a quantitative index evaluating the functional status of sciatic nerve, was calculated by the following formula (1) [32–34]. Interpretation of variables are as follows. The initial letter ‘E’ and ‘N’ means ‘experimental sides (E)’ and ‘normal sides (N)’ respectively. ‘PL’ is the abbreviation of ‘print length’, which is the distance from the heel to the third toe. ‘TS’ is the simplification of toe spread, which is the distance from the first to the fifth toe. ‘ITS’ is the initialism of intermediary toe spread, which is the distance from the second to the fourth toe. Combining the side (E, N) and the footprint length (PL, TS, ITS), EPL, ETS, EITS, NPL, NTS and NITS are got, which indicate the footprint length at specific side. Normally, index SFI is a negative number varying from − 100 to 0 (1) [32]. 0 is a good indicator of normal function of sciatic nerves, while − 100 suggests the severe injury of peripheral nerve. It was widely believed that the function of peripheral nerve was positively correlated to the SFI value. Red ink was used to dye foot of rats to keep track of natural footprints. For each rat, at least 4 integral footprints were collected and analyzed. SFI value was then calculated based on the recording.

1 $${\text{SFI}} = - 38.3\frac{{\left( {{\text{EPL}} - {\text{NPL}}} \right)}}{\text{NPL}} + 109.5\frac{{\left( {{\text{ETS}} - {\text{NTS}}} \right)}}{\text{NTS}} + 13.3\frac{{\left( {{\text{EIT}} - {\text{NIT}}} \right)}}{\text{NIT}} - 8.8.$$

**Fig. 6:**
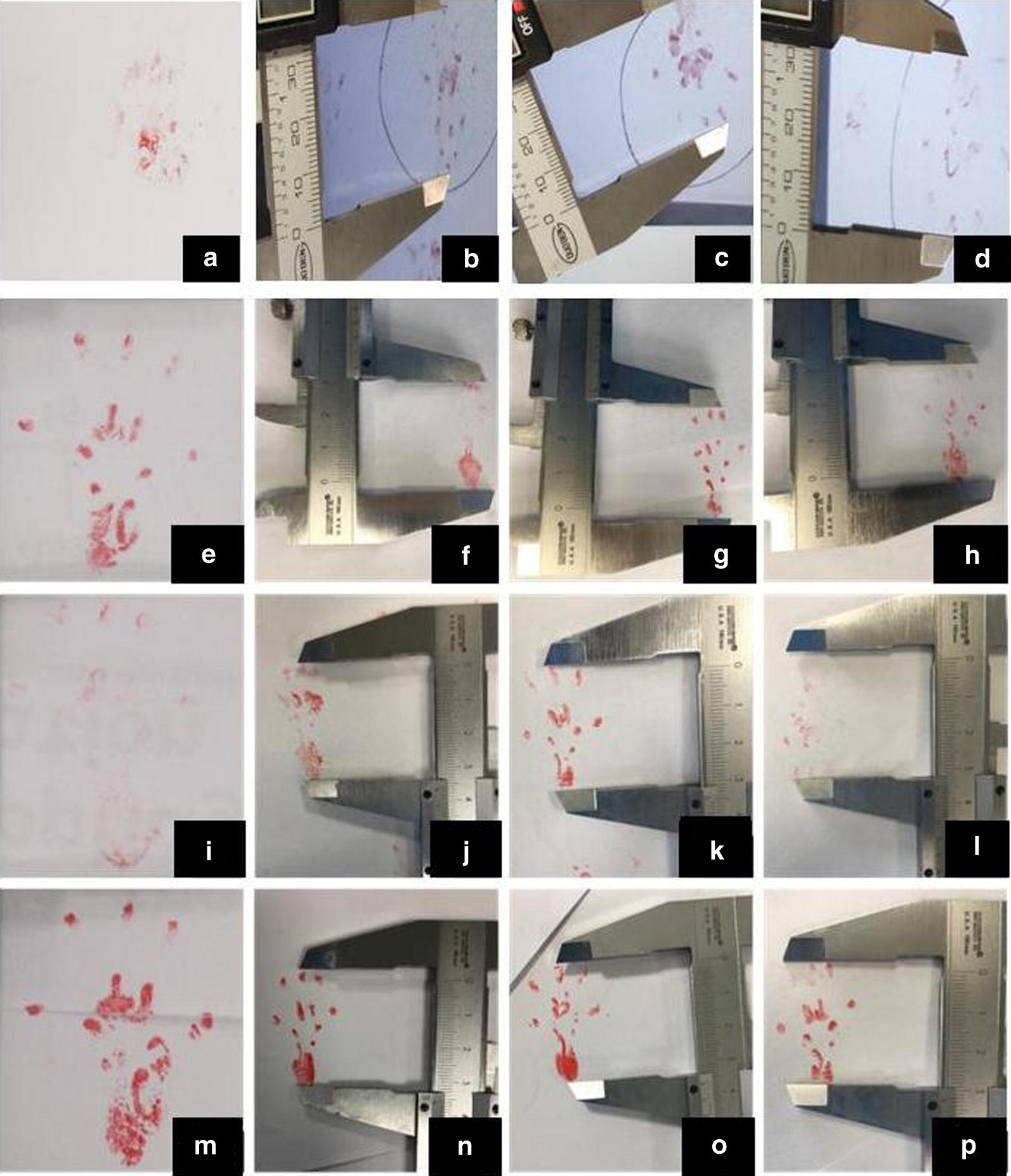
The footprint images of **a**–**d** PBS group, **e**–**h** VEGF group, **i**–**l** NGF group, and **m**–**p** VEGF + NGF group. Image **a**, **e**, **i**, **m** were normal side, and image **b**–**d**, **f**–**h**, **j**–**l**, **m**–**p**) are experimental side in corresponding groups

**Fig. 7:**
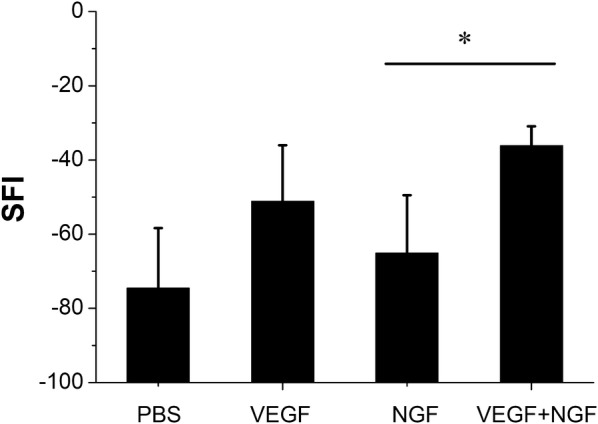
Index SFI of PBS, VEGF, NGF and VEGF + NGF groups at 4 weeks after surgery. *Indicates p < 0.05

Footprints of all rats were tracked on white paper by stained their foot with red ink. As can be seen in Fig. 6, normal sides (Fig. 6a, e, i, m) and experimental sides (Fig. 6b–d, f–h, j–l, n–p) were respectively sorted out. EPL, ETS, EITS, NPL, NTS and NITS were then measured to calculate SFI. Data were show in Fig. 7. From the result we could find that there is statistical difference between NGF group and VEGF + NGF group, which implies faster nerve recovery of mice in VEGF + NGF group. While for VEGF group, the therapeutic effect was similar to that in VEGF + NGF group (p > 0.05). Therefore, here we did not observe there exist biologically synergistic recovery effect between VEGF and NGF. However, we think it may be caused by the short term recovery time we used in this study (4 weeks), as it’s usually reported to last for 8 weeks or even longer for nerve recovery [8, 9].

### Histological analysis

**Fig. 8:**
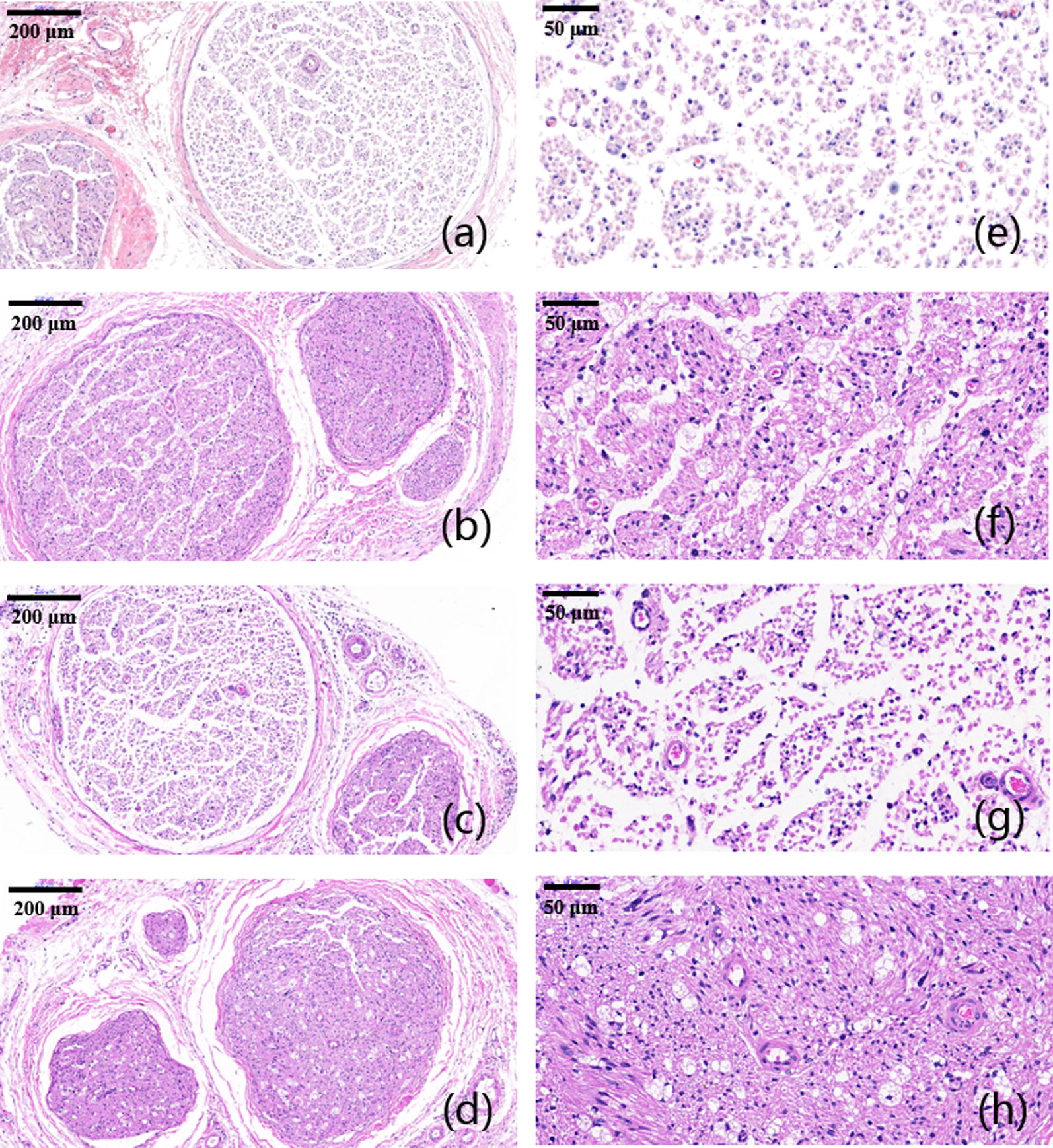
H&E staining of regenerated peripheral nerves at 4 weeks after surgery in **a**, **e** PBS group, **b**, **f** VEGF group, **c**, **g** NGF group, and **d**, **h** VEGF + NGF group. The scale bar is **a**–**c** 200 μm, and **d**–**f** 50 μm respectively

**Fig. 9:**
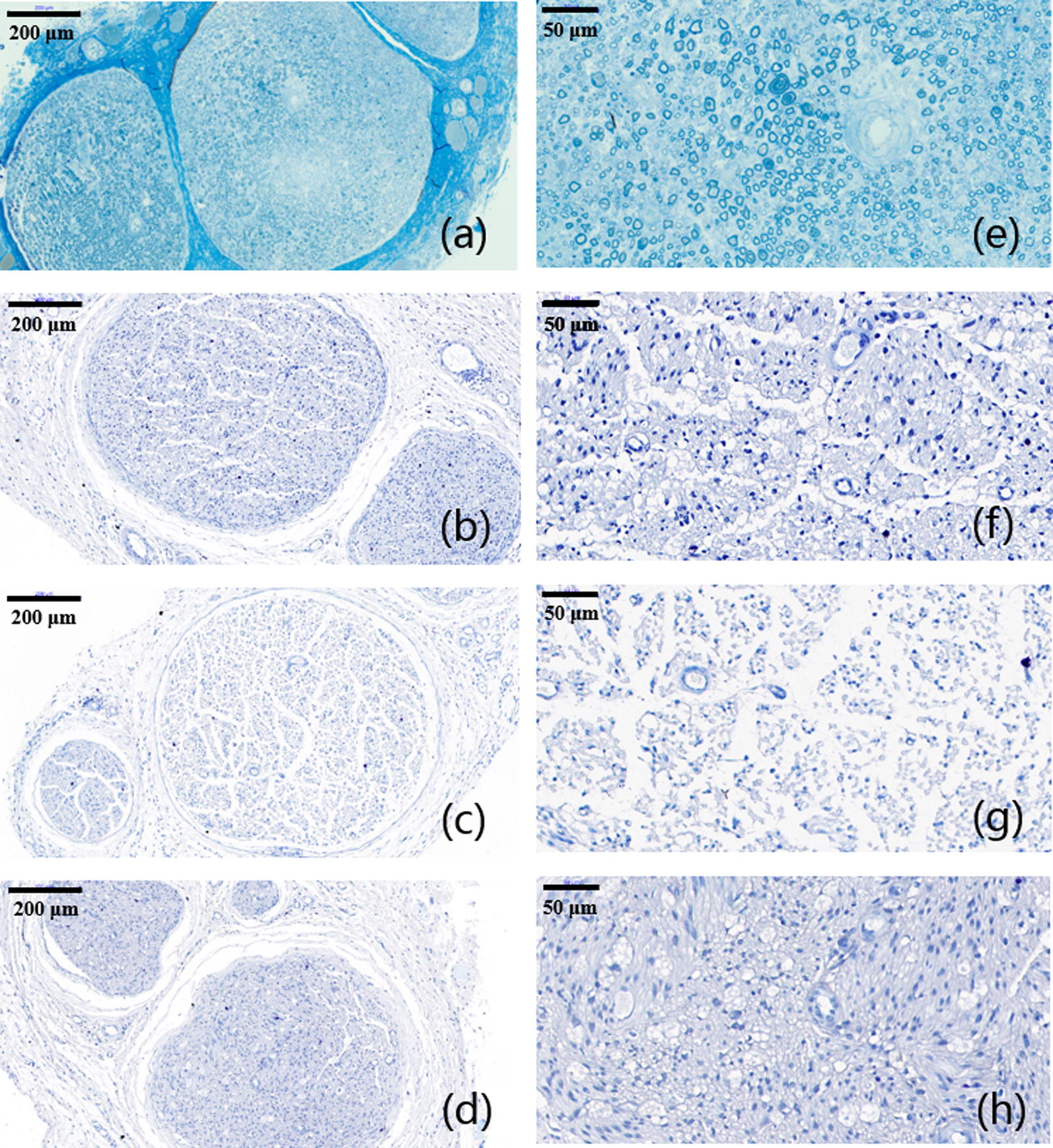
TB staining of regenerated peripheral nerves at 4 weeks after surgery in **a**, **d** VEGF group; **b**, **e** NGF group; **c**, **f** VEGF + NGF group. The scale bar is **a**–**c** 200 μm; **d**–**f** 50 μm respectively

**Fig. 10:**
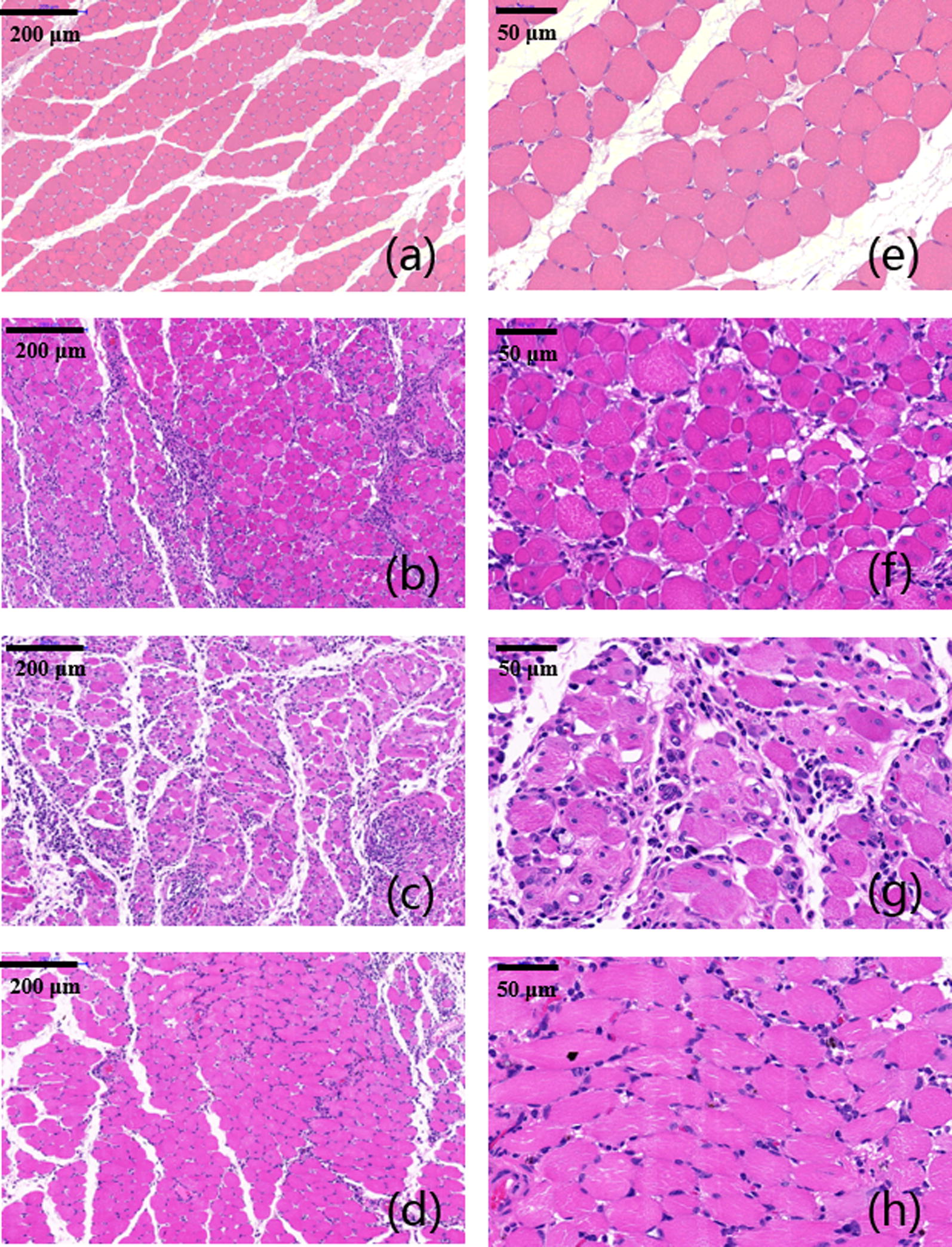
H&E staining of gastrocnemius muscles at 4 weeks after surgery in **a**, **e** PBS group, **b**, **f** VEGF group, **c**, **g** NGF group, and **d**, **h** VEGF + NGF group. The scale bar is **a**–**c** 200 μm, and **d**–**f** 50 μm respectively

**Fig. 11:**
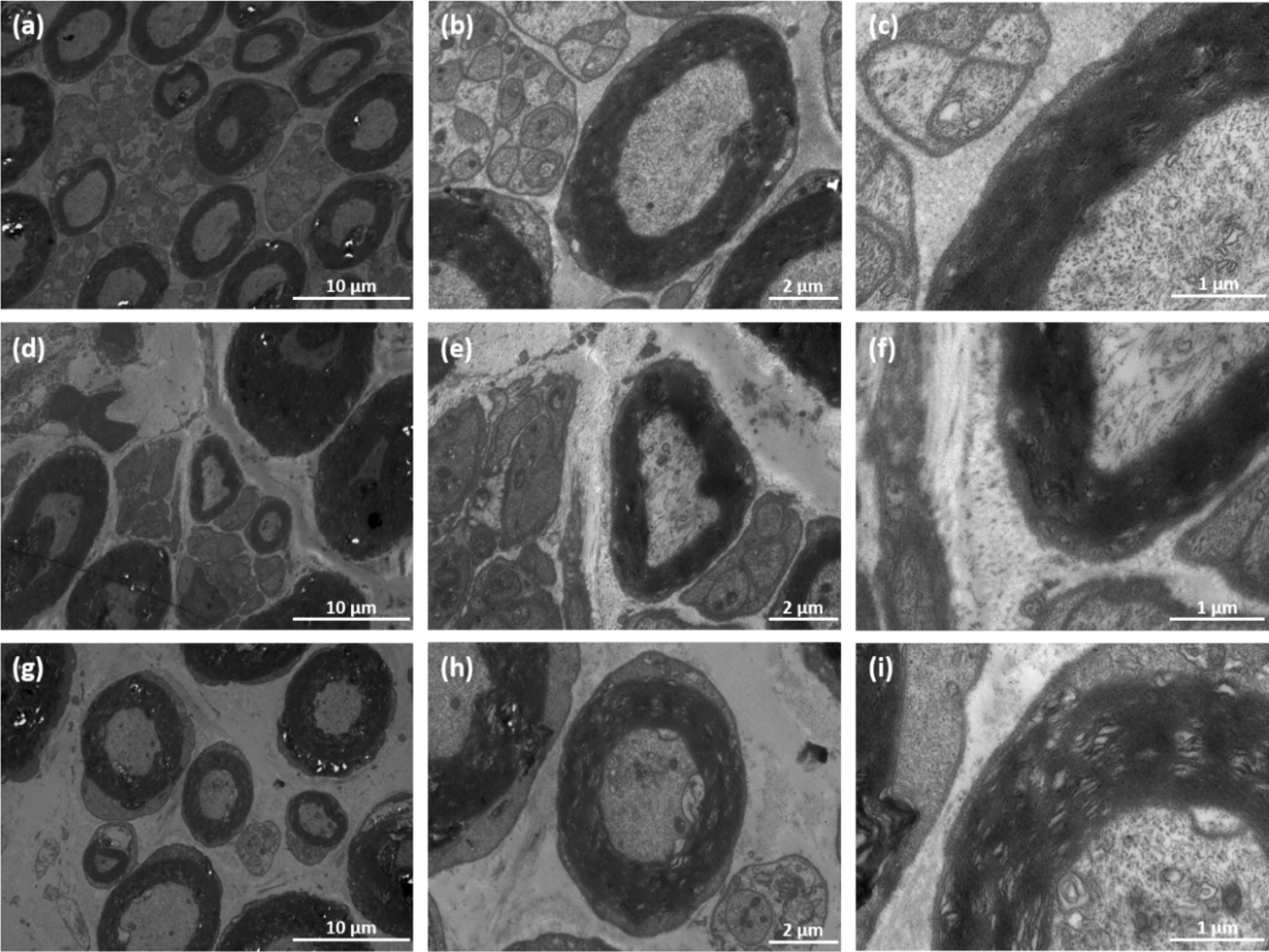
TEM of regenerated myelinated axons for ratsat 4 weeks after surgery in **a**–**c** VEGF group; **d**–**f** NGF group; **g**–**i** VEGF + NGF group. The scale bar is **a**, **d**, **g** 10 μm; **b**, **e**, **h** 2 μm; **c**, **f**, **i** 1 μm, respectively

**Fig. 12:**
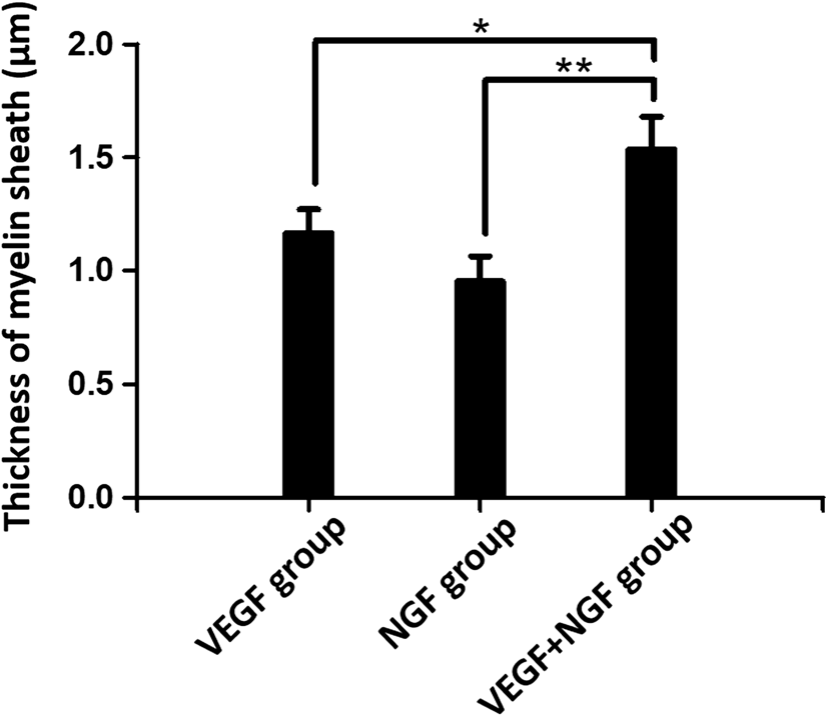
Thickness of myelin sheath for rats at 4 weeks after surgery in VEGF, NGF, and VEGF + NGF groups. *Indicates p < 0.05, **indicates p < 0.01

Immediately after the sacrifice, sciatic nerves of all rats were dissected. Besides, gastrocnemius muscles at the experimental side was removed and collected for further analysis. Dissected nerves were cut into 5 μm thick sections for further histological examination. Both toluidine blue (TB) staining and TEM scan were performed to evaluate the status of regenerated nerves. Hematoxylin and eosin (H&E) staining was performed as well to evaluate growth of muscle fibers. TB and H&E staining and TEM observation of sciatic nerves and gastrocnemius muscles were shown below (Figs. 8, 9, 10, 11). As we can see, most of the regenerated nerves in VEGF and VEGF + NGF groups were well organized, which indicates a great recovery in these groups. Semi-quantitative results on thickness of myelin sheath were shown in Fig. 12, which were measured and calculated based on TEM images. As can be seen, the thickness of myelin sheath in VEGF + NGF group is significantly higher than those in both VEGF and NGF groups, suggesting the optimal regeneration condition in VEGF + NGF group. This also implies us that there may exist a potential synergistic effect between VEGF and NGF.

### Assessment of angiogenesis

Regenerated sciatic nerves were dissected in the middle section, and the transverse layer was used for CD34 and DAPI double-immunofluorescence assays. The regeneration of microvessels in randomly selected views was used to evaluate the degree of angiogenesis.

**Fig. 13:**
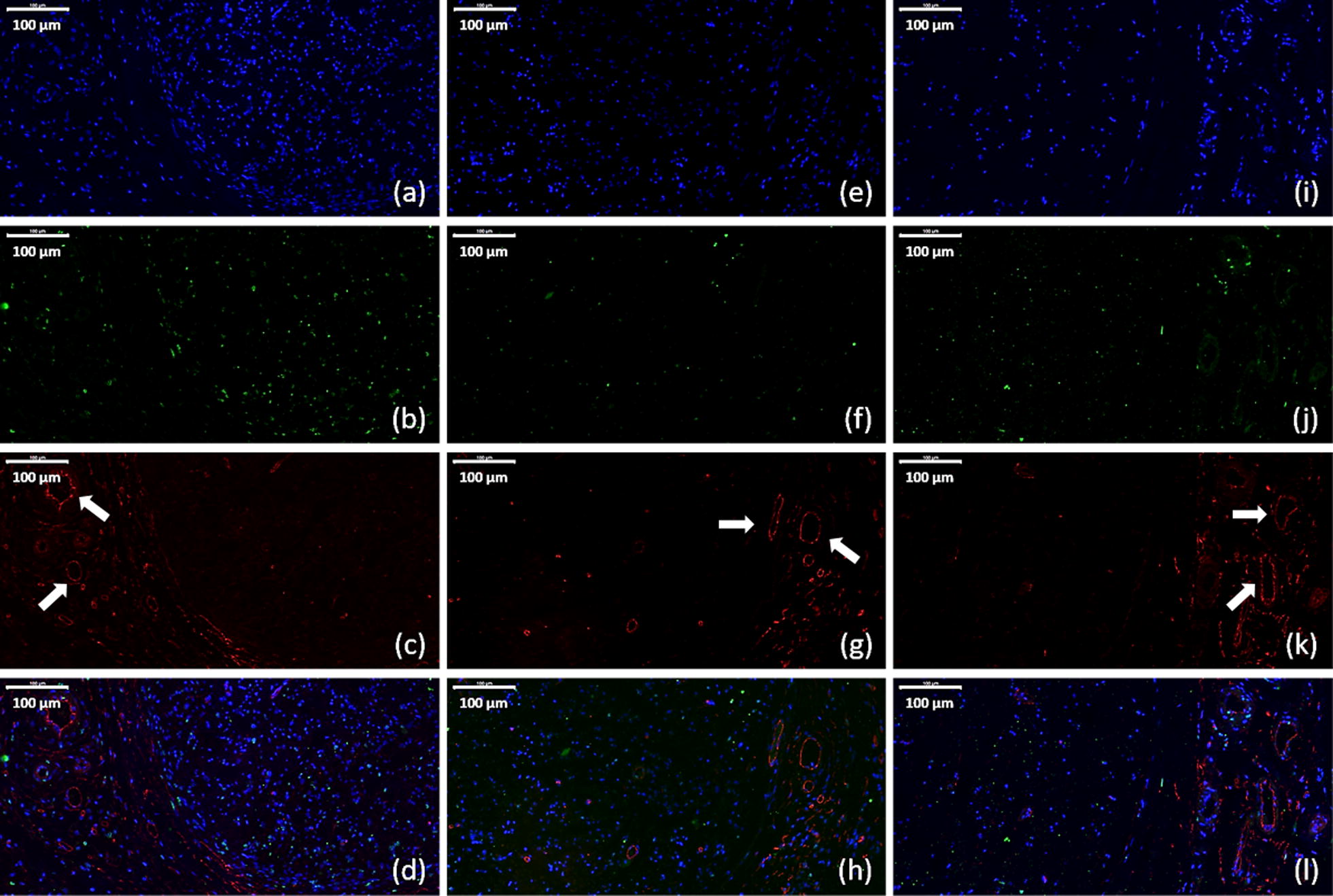
Immunofluorescence staining of regenerated peripheral nerves at 4 weeks after surgery in **a**–**d** VEGF group; **e**–**h** NGF group; **i**–**l** VEGF + NGF group. **a**, **e**, **i** DAPI staining (blue); **b**, **f**, **j** BrdU labeling (green); **c**, **g**, **k** CD34 staining (red); **d**, **h**, **l** merge. The scale bar is 100 μm

**Fig. 14:**
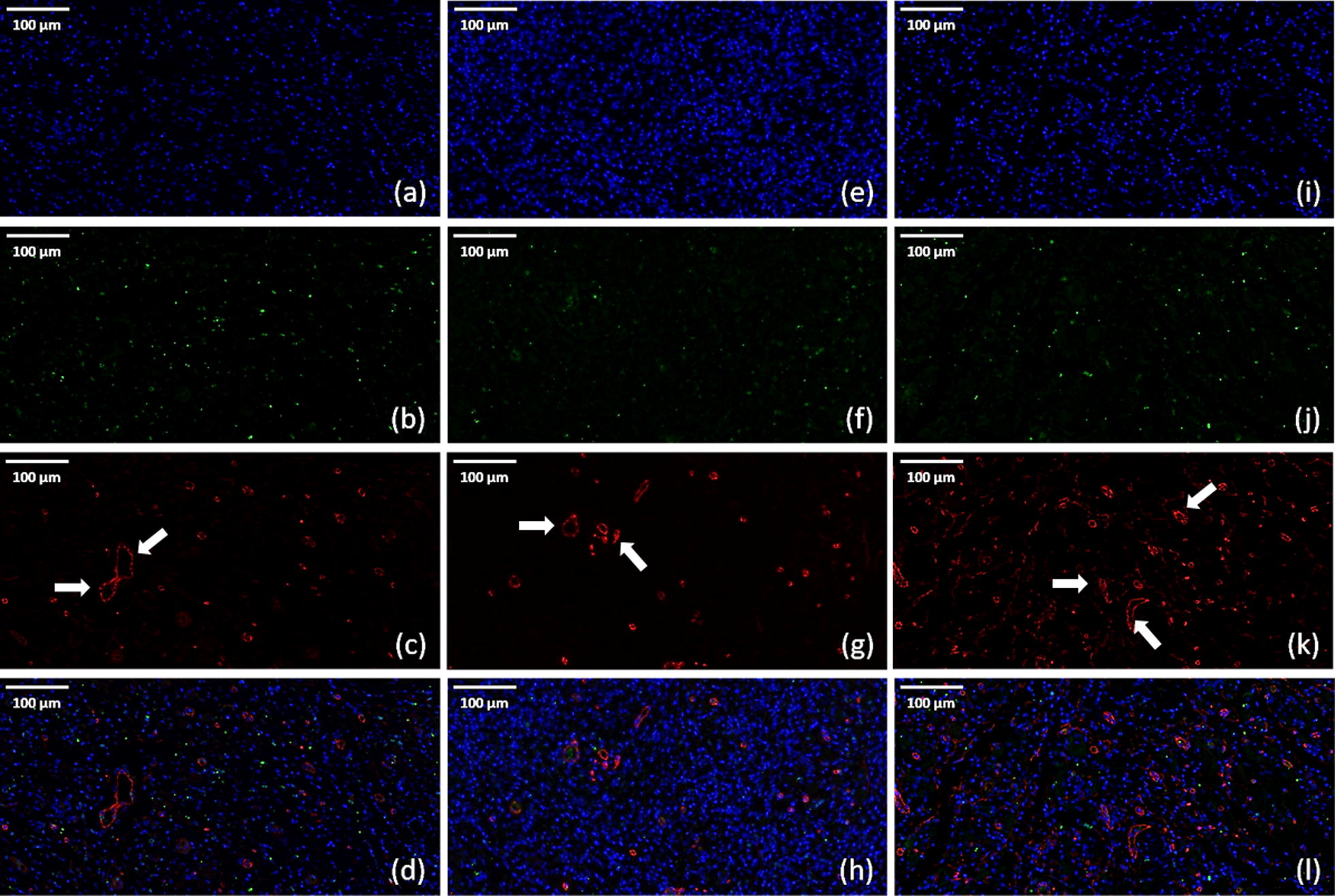
Immunofluorescence staining of gastrocnemius muscles at 4 weeks after surgery in **a**–**d** VEGF group; **e**–**h** NGF group; **i**–**l** VEGF + NGF group. **a**, **e**, **i** DAPI staining (blue); **b**, **f**, **j** BrdU labeling (green); **c**, **g**, **k** CD34 staining (red); **d**, **h**, **l** merge. The scale bar is 100 μm

To confirm that the advantageous performance in nerve recovery in VGEF + NGF group comes from the synergistic effect between NGF and VEGF, we then tested the angiogenesis levels in both regenerated nerves and gastrocnemius muscles in different groups. As can be seen in Figs. 13 and 14, all groups show proliferation of new cells after 4 weeks and some of them have regenerated microvessels in both peripheral nerves and gastrocnemius muscles. We could find that in regenerated peripheral nerves, both VEGF and VEGF + NGF groups show a high level of new cell proliferation, as shown in the BrdU staining results in Fig. 14b, f. Consistent with this results, regenerated microvessels in these two groups were also higher compared to that in NGF group (Fig. 13). While in regenerated gastrocnemius muscles, we found NGF group and VEGF group shows a similar effect in angiogenesis, and the density of microvessels was significantly higher in VEGF + NGF group (Fig. 14), which indicated an optimal regenerated condition for tissue recovery. Therefore, we could mention that the combination of VEGF and NGF could be a better strategy that could enhance the regeneration of sciatic nerve and peripheral muscle tissues.

## Discussion

Cationic polymer could be used as a vestile vector for the delivery of gene materials. In this work, we use PDAPEI polycation to condense VEGF and NGF for the treatment of peripheral nerve injury. It is obviously that with the increase of the w/w ratio of PDAPEI to pDNA, the transfect efficient was improved. This is probably because that the positive charges on the surface of the polyplex was relative high and could enhance the endocytosis of these nanoparticles. However, the cytotoxicity of the polyplex was also increased due to the larger using amount of the polycation. So we should balance the transfect efficiency and cytotoxicity of the polyplex and optimize the formulation when using this polymer as gene carrier.

## Conclusions

Generally, combining these experiment results above, we confirmed the condensing ability of synthetic cationic polymer PDAPEI to form polyplex nanoparticles. We utilized this biodegradable polymer to deliver VEGF and NGF in a combinational way for the treatment of peripheral nerve injury. A conclusion could be made that PDAPEI/pDNA polyplexes at 30 w/w ratio in our study showed a good performance in gene delivery. This non-viral carrier delivering VEGF and NGF in a combinational way is a promising strategy for the peripheral nerve regeneration.

## Methods

### Materials

Branched PEI (molecular weight of 1.8 k and 25 kDa), and dimethylformamide (DMF) were obtained from Sigma-Aldrich. 2,6-pyridinedicarboxaldehyde (PDA) was obtained from TCI (Shanghai) Development Co., Ltd. Cellulose membranes (MWCO = 10 kDa), Dulbecco’s modified Eagle’s medium (DMEM), phosphate buffered saline (PBS, pH 7.4 basic) and fetal bovine serum (FBS) were purchased from Thermo Fisher Scientific (Shanghai, China). Rat Schwann cells (RSC) was bought from the cell bank of the Chinese Academy of Sciences (Shanghai, China).

### Synthesis of PDAPEI polymers

Similar procedures were used in synthesis of PDAPEI polymers as described in our previous work [18, 19]. In brief, 2 mmol PDA dissolving in 20 mL DMF were dropwise added into 20 mL DMF containing 1 mmol PEI (1.8 kDa) with vigorous stirring. The reaction took 48 h at room temperature before the mixture was dialyzed through the cellulose membrane (MWCO = 10 kDa). The terminal yellowish product PDAPEI were harvest through lyophilization, and were kept at − 20 °C for future use.

### Preparation of PDAPEI/pDNA polyplexes

Briefly, plasmid DNA (pDNA) solutions were diluted to 20 ng/μL by deionized water. Polycationic material PDAPEI was dissolved in water at the concentration of 2 mg/mL. pDNA solution (20 ng/μL) were added into PDAPEI solutions with predetermined concentrations and incubated at room temperature to form PDAPEI/pDNA polyplexes with different mass ratios (weight/weight, w/w). Final concentrations of pDNA in these polyplex samples were all kept at 10 ng/μL. Polyplexes were preserved at room temperature for at least 30 min for further experiments or characterizations.

### Morphology observation

Morphology of polyplexes was observed by Transmission Electron Microscopy (TEM, JEOL JEM 2010 system) under 120 kV. 10 μL polyplex solution was taken out carefully to be added on the copper net and then incubated overnight under room temperature. Polyplexes were observed by transmission electron microscopy (TEM) and images were recorded for further analysis.

### In vitro cell cytotoxicity of PDAPEI/pDNA polyplexes

The cell cytotoxicity of PDAPEI/pDNA polyplexes on Rat Schwann cells (RSC) were evaluated by CCK-8 reagent. RSC were cultured in 96-well plates with 50 µL DMEM complete growth medium at 37 °C in a 5% CO_2_ humidified atmosphere. When the confluency reach 70–80%, 10 µL PDAPEI/pDNA polyplexes at various w/w ratios were transfected into cells for 4 and 24 h respectively and then evaluated with CCK-8 reagent.

### In vitro cell transfection

In this study, transfection effciency of PDAPEI/pDNA polyplexes was evaluated on RSC. RSC were seeded in 24-well plate with 1000 µL high-glucose DMEM supplemented with 10% FBS and incubated at 37 °C in a 5% CO2 humidified atmosphere. When each well was 70–80% confluent, 100 µL PDAPEI/pDNA polyplexes at various ratios, PEI (25 kDa)/pDNA polyplexes as positive control group, were transfected into cells for 4 h in 500 µL fresh DMEM. Afterwards, the medium was replaced by 1000 µL DMEM complete growth medium and incubated for additional 72 h. Then cells were detached by trypsin for evaluating GFP expression level by flow cytometer.

### Peripheral sciatic nerve crush injury model

Peripheral sciatic nerve crush injury model was established on Sprague Dawley (SD) rats to evaluate the therapeutic effect of PDAPEI/pDNA polyplexes. All surgical operations followed instructions in published studies [8, 9, 35, 36]. Briefly, SD rats (weight of 150 ± 10 g) were maintained in the SPF environment for 1 week and then anesthetized by 10% chloral hydrate (0.3 mL/kg) via intraperitoneal injection. Body temperature of rats during the operations was maintained around 37 °C. Furs on posterior thigh and gluteal regions were carefully shaved with electric clippers, and then the skin was exposed and cleaned using sterile cotton applicators. Afterwards, a semi-circular 3 cm incision across midline was made longitudinally in the skin, and the skin was dissected from the underlying musculature gently. The sciatic nerve was exposed through gluteal splitting approach, and further freed from the surrounding connective tissues with soft tissue retractors and iridectomy scissors. After that, the middle part of sciatic nerve is crushed by 5-inch hemostatic forceps for 15 s at 3 clicks. Finally, all rats were sutured and injected intraperitoneally with appropriate amount of ampicillin. The rats were taken good care of in the first week after operation, since the postoperative period was a very stressful time for both rats and operators. All rats were randomly allocated into four groups, with each group 3 rats: PBS group, VEGF group, NGF group and VEGF + NGF group. Two days after surgery for skin would healing, drugs were injected every 2 days. For treatment groups, 0.3 mL polyplex solution containing 30 µg total pDNA was intramuscularly injected to the surgical site. All rats were sacrificed after 4 weeks. The peripheral sciatic nerve crush injury model was established in this study. In this study, all animals were performed according to the guidelines approved by the Institutional Animal Care and Use Committee of Shanghai Jiao Tong University (SJTU, No. A2017073).

### Statistical analyses

All tests were repeated independently and data were shown as mean ± standard deviation. p < 0.05 was regarded as significant using GraphPad Prism 7 software (GraphPad Prism, Inc). Significant differences were measured using one-way analysis of variance (ANOVA) among groups. Significant differences were evaluated using two-tailed t test between two groups.

## Data Availability

All data supporting this study are included in this article.

## Abbreviations

pDNA: Plasmid DNA
bPEI: Branched polyethylenimine
VEGF: Vascular endothelial growth factor
NGF: Nerve growth factor
Mw: Weight average molecular weight
GPC: Gel permeation chromatography
TMS: Tetramethylsilane
H^1^-NMR: Proton nuclear magnetic resonance
SD: Sprague–Dawley
H&E: Hematoxylin and eosin
TB: Toluidine blue
DMEM: Dulbecco’s modified Eagle’s medium
PBS: Phosphate buffered saline
FBS: Fetal bovine serum
PDA: 2,6-Pyridinedicarboxaldehyde
DMF: Dimethylformamide
RSC: Rat Schwann cell
FTIR: Fourier transform infrared spectrum
PDI: Polydispersity index
PEG: Polyethylene glycol
DAD: Diode array detector
RID: Refractive index detector
TEM: Transmission Electron Microscopy
EDTA: Ethylene diamine tetraacetic acid
TAE: Tris-Acetate-EDTA
